# Cancer immunomodulation using bispecific aptamers

**DOI:** 10.1016/j.omtn.2022.01.008

**Published:** 2022-01-10

**Authors:** Brian J. Thomas, David Porciani, Donald H. Burke

**Affiliations:** 1 Department of Molecular Microbiology and Immunology, University of Missouri School of Medicine, Columbia, MO 65212, USA; 2 Bond Life Sciences Center, University of Missouri, Columbia, MO 65201, USA

**Keywords:** bispecific, oligonucleotide therapy, antibody, immunomodulation, oncolytic, molecular design, clinical translation

## Abstract

Evasion of immune destruction is a major hallmark of cancer. Recent US Food and Drug Administration (FDA) approvals of various immunomodulating therapies underline the important role that reprogramming the immune system can play in combating this disease. However, a wide range of side effects still limit the therapeutic potential of immunomodulators, suggesting a need for more precise reagents with negligible off-target and on-target/off-tumor effects. Aptamers are single-chained oligonucleotides that bind their targets with high specificity and affinity owing to their three-dimensional (3D) structures, and they are one potential way to address this need. In particular, bispecific aptamers (bsApts) have been shown to induce artificial immune synapses that promote T cell activation and subsequent tumor cell lysis in various *in vitro* and *in vivo* pre-clinical models. We discuss these advances here, along with gaps in bsApt biology at both the cellular and resident tissue levels that should be addressed to accelerate their translation into the clinic. The broad application, minimal production cost, and relative lack of immunogenicity of bsApts give them some ideal qualities for manipulating the immune system. Building upon lessons from other novel therapies, bsApts could soon provide clinicians with an immunomodulating toolbox that is not only potent and efficacious but exercises a wide therapeutic index.

## Graphical abstract

**Figure:**
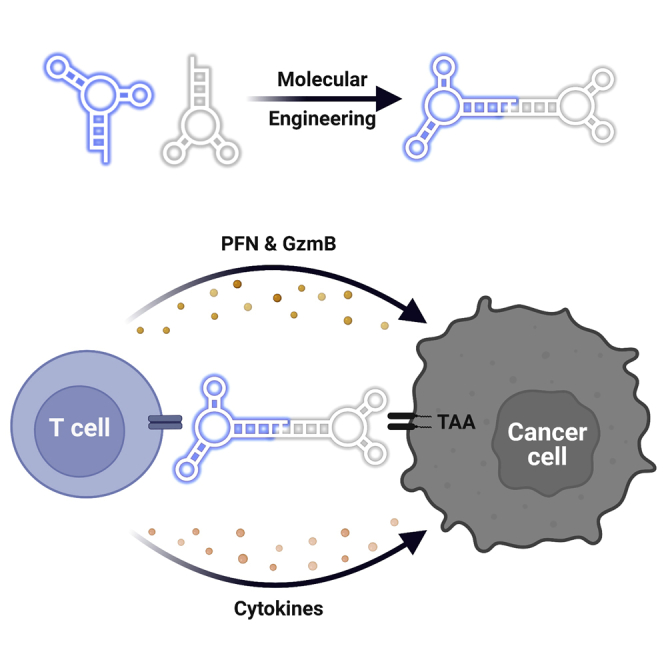

## Teaser

Bispecific therapeutics that dually target cell surface receptors on immune cells and cancer cells have proved effective in the treatment of cancer. Using oligonucleotides to develop such reagents has many advantages, such as ease of manipulation and low immunogenicity. With careful evaluation, these reagents could soon provide an alternative or adjuvant therapy for difficult-to-treat cancers.

## Introduction

### Cancer as a foreign body

Cancer is the second leading cause of death globally and is expected to surpass cardiac disease as the number one cause of death in the next few years, if it has not done so already.^1^, ^2^, ^3^ Underlying the majority of cancers are common biological pathways that, when transformed, lead to sustained proliferative signaling and dysregulated cellular energetics. Non-lethal genetic damage is the defining transforming factor of carcinogenesis, in that an initiating mutation leads to the accumulation of complementary mutations, ultimately leading to genomic instability and acquisition of various other cancer hallmarks.^4^^,^^5^ One such hallmark, the evasion of immune destruction, has recently become the center of attention for novel therapeutics to eradicate the body of clonally derived cancer cells.^6^ Conventional methods used to address cancer eradication include chemotherapy, radiation, and surgery. Chemotherapy and radiation are both mainstays of treatment for most hematologic and late-stage cancers. However, due to their lack of specificity, these therapies often come with profound side effect profiles (e.g., anthracycline-induced cardiotoxicity) and significant risks for secondary malignancy (e.g., radiation-induced thyroid carcinoma). Resection, or surgical removal, is typically reserved for early-stage solid tumors, and setbacks occur when undetected malignant cells are left behind. Newer therapies, such as biologics and small molecule inhibitors, continue to emerge and address some of the off-target (i.e., non-specific) effects of conventional treatments.^7^^,^^8^ While these therapies are targeted, they too have their own side effect burdens, and in most cases drug resistance occurs within a few months to years.^9^^,^^10^ There is an ongoing need to expand our therapeutic toolbox with more efficacious therapies that are more specific, longer lasting, and have lower side effect profiles.

To address this need, much attention has been given to using biologics to redirect the immune system to improve recognition of tumors and to prevent their growth or relapse.^6^ The intention of such re-directive therapies is not only to help the immune system recognize cancer cells as foreign but also to induce an eradication response and provide long-term memory. Indeed, the immune system often does initially recognize cancer cells as foreign, as it does for bacteria, viruses, parasites, and fungi. However, cancer cells employ mechanisms to evade immunosurveillance, such as downregulating major histocompatibility complex (MHC)-mediated antigen presentation, releasing proteins to induce lymphocyte apoptosis (e.g., Fas ligand [FasL], tumor necrosis factor alpha [TNFα]), releasing immunosuppressive cytokines (e.g., interleukin [IL]-10, transforming growth factor beta [TGFβ]), attracting immunosuppressive cell populations (e.g., regulatory T cells [Tregs], myeloid-derived suppressor cells [MDSCs]), upregulating cell surface molecules that block T cell activation (e.g., programmed death-ligand 1 [PD-L1]), or downregulating co-stimulatory molecules to evade immune detection (e.g., cluster of differentiation [CD] 80/CD86).^5^^,^^11^ Weak or ineffectual tumor-suppressive immune responses then enable cancers to emerge and thrive. Preventing these evasive regulatory mechanisms with genetic modifying agents such as RNA interference (RNAi) and redirecting immune cells through immune checkpoint inhibiting (ICI) antibodies (Abs) are examples of how biologics are being used for cancer immunomodulation. Because cancer cells are derived from host cells, they were once “familiar” to immune defenses, and they often exhibit few or no distinct features that identify them as non-self. This self-like property makes the discovery of uniquely cancer-specific antigens exceedingly difficult. Nevertheless, the composition of cellular machinery in cancer cells does differ from that of the homeostatic cell population. These compositional differences, hereafter referred to as tumor-associated antigens (TAAs), can be subtle or profound depending on the cancer type, and they are the rationale behind many of the newest immunomodulating therapies such as chimeric antigen receptor (CAR) T cells and bispecific antibodies (bsAbs). Because of the mechanistic variations in these therapies, it is important to note that immunomodulation can be achieved and defined in various ways (Table 1).

**Table 1:** Defining immunomodulation terms

Term	Action	Consequence
T cell activation	granzyme and perforin release	tumor cell lysis
	FasL or TNFα release	tumor cell lysis
	pro-inflammatory cytokine production	various
Co-stimulation	secondary signal	induce T cell activation
Co-inhibition	secondary signal	prevent T cell activation
Co-potentiation	conformational change	lower threshold for induction of TCR signaling by pMHC
Silent cell-cell junction	None	bring two cells into close proximity
Artificial immune synapse	induce artificial receptor signaling (e.g., TCR, co-receptor)	bring two cells into close proximity and induce T cell activation (provide signals one or two)
T cell memory	effector (peripheral; CD44+/CD62L-) or central (CCR7+/CD62L+) anti-cancer response	prevent cancer recurrence

TCR, T-cell receptor; pMHC, antigen or peptide presented on major histocompatibility complex; CD, cluster of differentiation.

A central goal of cancer therapeutic development has been to shift biodistribution to favor delivery exclusively to cancer cells, away from non-tumor tissues, thereby precluding many of the off-target or on-target/off-tumor effects. The latter happens when a targeted anti-cancer reagent recognizes its target marker (on-target) on healthy cells (off-tumor) that have above-threshold expression levels of the same marker, ultimately resulting in severe cytotoxicity or undesired detection. While newer therapies have had great initial successes, a plethora of reported side effects limit their dosing and effectiveness.^12^ Immunomodulatory therapies in particular can cause potentially lethal immune-related adverse events (irAEs) such as excessive cytokine release, neurotoxicity, and tissue non-specific immune cell damage.^13^, ^14^, ^15^ This highlights the evolving need for directed therapies that have a higher therapeutic index and that allow for maximum cancer eradicating efficacy.

### Aptamer discovery and manipulation

Aptamers are emerging as tools for addressing this evolving need. Aptamers are short DNA and RNA oligonucleotides with three-dimensional (3D) structures that bind to targeted proteins with relatively high specificities and affinities, analogous in some respects to monoclonal antibodies (mAbs). In the past decade, we and others have widely exploited aptamers for use in biomedical science (single molecule imaging, *in vivo* imaging), personalized diagnostics, and targeted delivery of therapeutics.^16^, ^17^, ^18^, ^19^, ^20^, ^21^, ^22^, ^23^, ^24^ Aptamers possess many properties that can be considered comparable with or even advantageous relative to mAbs (Table 2),^25^, ^26^, ^27^, ^28^ perhaps the most important of which is the nearly complete lack of immunogenic responses that target aptamers as foreign molecules. This makes aptamers ideal candidates for use as immunomodulatory agents. Nevertheless, aptamers have yet to be widely accepted for clinical use, suggesting there are still substantial barriers to overcome. This review identifies areas in which immunomodulating aptamers are performing well and have potential for clinical translation, along with gaps in bsApt biology at both the cellular and resident tissue levels that should be addressed to accelerate their translation into the clinic.

**Table 2:** Aptamers possess many properties that are comparable with or advantageous over antibodies

	Aptamer	Antibody (mAb)
Comparable or advantageous		

Size	∼3 nm; <30 kDa higher tissue penetration	∼10–15 nm; ∼150 kDa lower tissue penetration
Target	Immunogenic and non-immunogenic targets	Only immunogenic targets
Cost	Low	High, requires animal model^a^
Synthesis	Synthetic; bench top (*in vitro*)	Physiologic^a^; animal model (*in vivo*); labor intensive
Batch-to-batch variation	Minimal or none	Yes
Scalability	Yes	Minimal
Stability (pH, temp)	High; ability to refold in the proper 3D structures upon denaturation	Low or moderate
Shelf life	Months (room temperature) to years (frozen)	Months (refrigeration) to years (frozen)
Immunogenicity (i.e., immune response to foreign material)	Minimal or none	High (less if humanized)
Conjugation	Easy	Difficult
Other post-production modification	Easy	Difficult
Specificity	High	High
Affinity	High (nM to pM)	High (nM to pM)
Molecular forces involved in binding	Electrostatic, hydrogen bonding, hydrophobic interaction, van der Waals	Electrostatic, hydrogen bonding, hydrophobic interaction, van der Waals
Internalization via endocytosis	Minimal	Minimal or absent
Diagnostic use	Yes	Yes

Disadvantageous or unknown		

Half-life	Low (minutes to hours); renal excretion due to small size; nuclease susceptible^b^	High (weeks); FcRn receptor recycling
Nuclease susceptibility	Yes^b^ (less if modified)	None
Dissociation rate	Fast (due to monovalent nature)	Slow (due to bivalent nature)
Clinically accepted therapeutic use	Minimal (only one FDA approved)	Yes

Room temperature is ∼20°C.

Stylistic formatting for table contents need to be kept the same. Right now there is a mix of capitalizations (‘Low’, ‘yes’, ‘Yes’, ‘Easy’, ‘fast’). We prefer to use capitalization, but either way is fine.

a Exception: phage display.

b dependent upon chemical modifications.

**Figure 1:**
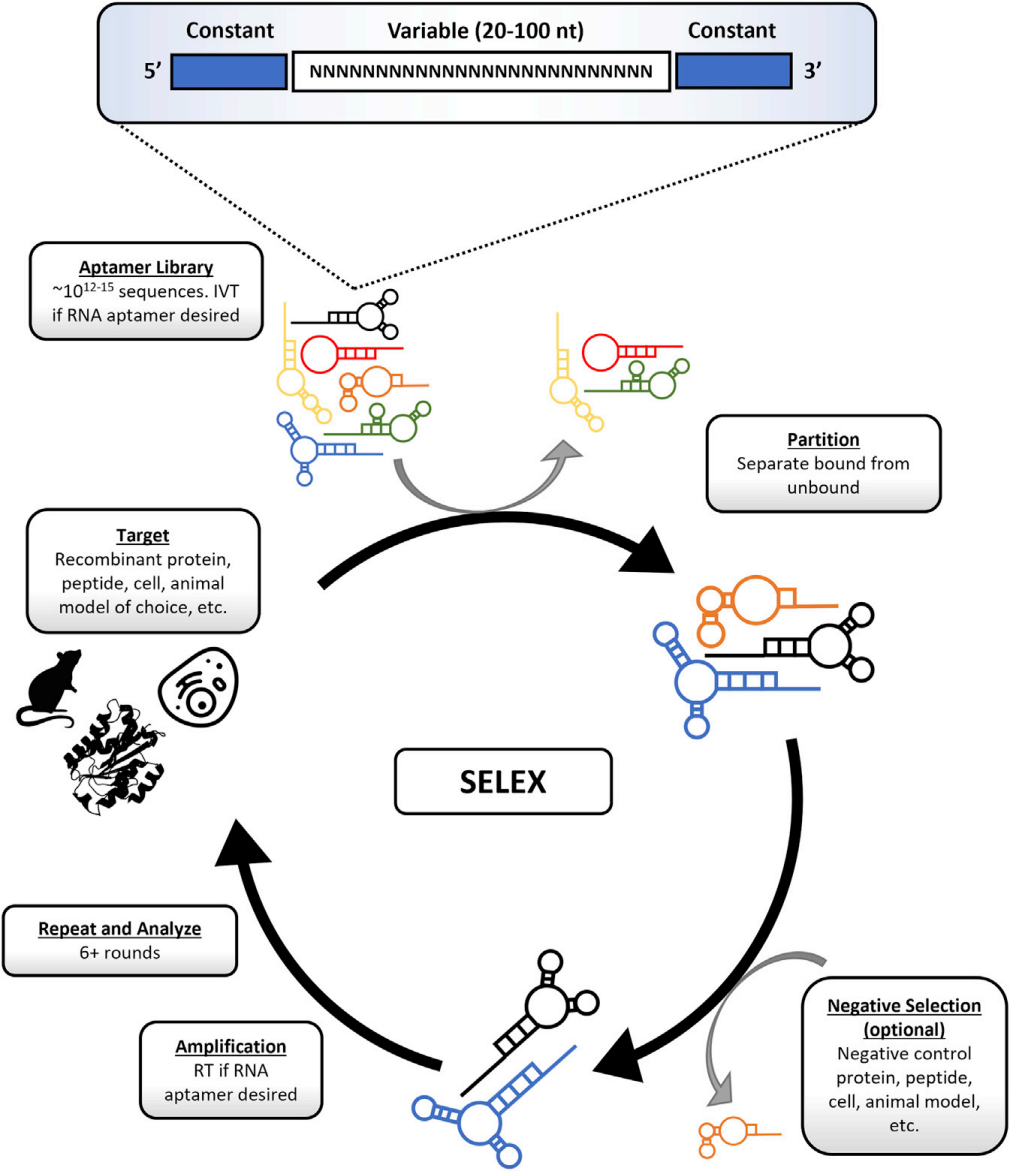
Aptamer selection (SELEX) Simplified overview of the aptamer selection process using SELEX. Starting with the box labeled “Target” and moving clockwise. Target: choose a target of interest, most commonly a recombinant protein but can be a peptide, cell line, or animal model. Aptamer Library: library consisting of ∼10^12−15^ sequence of DNA or RNA oligonucleotides. Library sequences are flanked by 5′ and -3′ constant regions called primer binding sites. If RNA oligonucleotides are desired, *in vitro* transcription (IVT) can be done prior to the next step. Partition: library is incubated with target of interest and species that are unbound are removed via multiple wash steps. Bound sequences are then eluted for the next step. Negative Selection: subtractive step using a control protein, peptide, cell line, or animal model. There is typically at least one negative selection per protocol. Amplification: the library is amplified via PCR. If RNA oligonucleotides were used, an additional reverse transcription (RT) step is required prior to amplification. Repeat and Analyze: the partitioning and optional subtraction steps are repeated 6–15 more times. The library is then sequenced and analyzed using various computational and laboratory methods.

**Figure 2:**
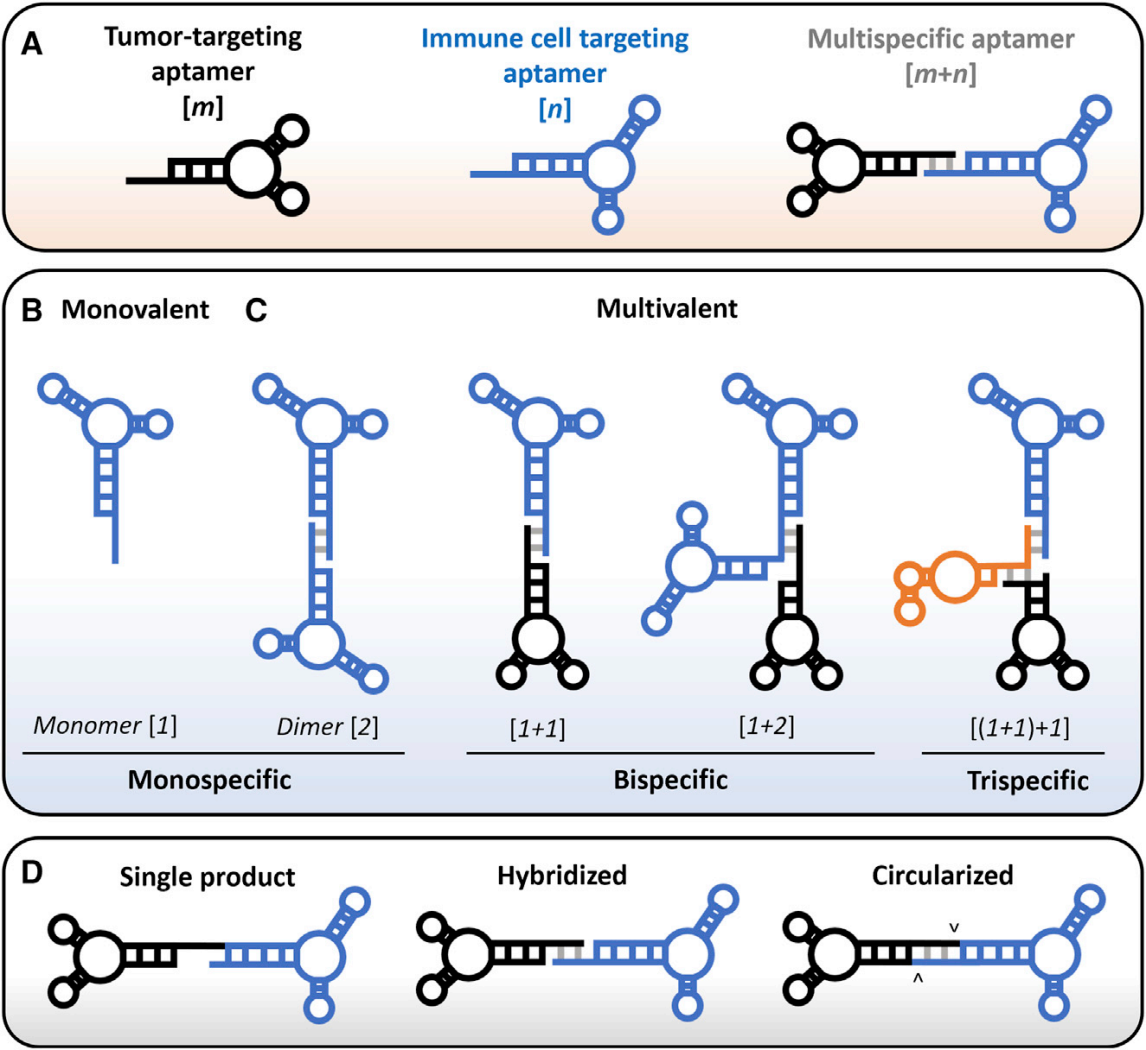
Defining valency and specificity (A) Multispecific aptamers can be characterized using the [*m* + *n*] nomenclature where the [*m*] represents the tumor-targeting aptamer and [*n*] represents the immune-cell-targeting aptamer. (B and C) Immunomodulating aptamers may be defined by valency (monovalent or multivalent) or specificity (monospecific or multispecific). Multivalent aptamers can bind the same cell (in *cis*) or two different cells (in *trans*). Immunomodulating trispecific aptamers have not yet be described in the literature but can follow [*m* + *n*] nomenclature wherein *m* or *n* is expanded by parenthesis. For example, a trispecific reagent that embodies two aptamers that bind two different cancer cell targets ([*m*] = 1 + 1) and one aptamer that bind one immune cell target ([*n*] = 1) would be denoted as [(*1* + *1*) + *1*]. (D) bsApts can by synthesized as a single product or as two separate products that are hybridized. These constructs may then be circularized by enzymatic or chemical ligation. Gray denotes hybridized bases. Arrowheads denote ligation sites.

Aptamer selection and development have been extensively reviewed elsewhere and are summarized in Figure 1.^24^^,^^29^, ^30^, ^31^ A great benefit of aptamers is their ease of chemical manipulation and molecular engineering, both prior to and after they have been selected.^27^^,^^32^, ^33^, ^34^, ^35^ Chemical modifications are often included to improve stability against serum nucleases, such as using 2'fluoropyrimidines (2′FY) or 2′-*O*-methyl pyrimidines (2′-OMeY) in place of natural 2′OH nucleotides, or to increase target binding affinity, such as the addition of hydrophobic or charged moieties on pyrimidine C5. Some of the major pre- and post-systematic evolution of ligands by exponential enrichment (SELEX) manipulations are summarized in Table 3. A post-SELEX manipulation that is especially relevant for this review is the generation of multivalent aptamers. Combining aptamers allows for simultaneous recognition of either two or more different cell surface receptors (bispecific aptamers [bsApts]) or multiple copies of the same receptor (dimeric, monospecific aptamers) (Figure 2). Furthermore, the targeted receptors can be on the same cell (in *cis* binding) or on two different cells (in *trans* binding). In the discussion of immunomodulating aptamers, multivalency and bispecificity enable physical conjugation of cancer cells and immune cells via an induced synapse. In addition, an attractive feature of bsApts is that the formation of a physical complex that connects different aptamers together could confer novel functionalities such as agonistic or antagonistic activities that are not displayed by the mixtures of the parental, monospecific aptamers. For example, in the case of bsApts used in cancer immunomodulation, this newly acquired activity can promote tumor cell lysis.

**Table 3:** Manipulation of aptamers pre and post-SELEX

	Example(s)	Outcome
Pre or post SELEX		

Phosphodiester modification	phosphorothiate linkage (i.e., thioaptamer)	decrease nuclease susceptibility; promote protein binding in plasma and tissue
Nucleobase modification	hydrophobic, aromatic, and charged moieties on pyrimidine C5 (e.g., SOMAmers, dDs-dPx)	increase binding affinity
Ribose substitution	2′FY, 2′ OMe, 2′NH2, LNA	decrease nuclease susceptibility

Post SELEX		

Truncation	removal of non-essential nucleotides	increase binding affinity
Combination	hybridization of complementary bases or covalent conjugation	multivalent aptamer (e.g., bispecific)
Crosslinkers	5-IdU, phenyl azide, or diazirine (UV activatable)	efficient pulldown of target proteins
5′ or 3′ modification	5′ PEGylation, cholesterol, DAG 3′ IdT, biotinylation	limit renal excretion and enhance serum half-life

Ds, 7-(2-thienyl)-imidazo[4,5-b] pyridine; dPx, 2-nitro-4-propynylpyrrole; IdU, iododeoxyuridine; LNA, locked nucleic acid; NH_2_, amine; DAG, dialkylglycerol; IdT, inverted deoxythymidine.

In this review, we first introduce immunomodulating monovalent and monospecific therapies such as mAbs and aptamers. Next, we focus our attention on immunomodulating multivalent therapies, with emphasis on bsApts, identifying features for effective aptamer multimerization and immunomodulation that have emerged from the most promising reagents. Finally, we provide a perspective on the opportunities that lie ahead to improve efficacy and safety of immunomodulating bsApts.

## Aptamers and related technologies used in immunomodulation

The major successes of ICIs and CAR T cells underline the power of appropriately recruiting the immune system in cancer therapy, often as an adjuvant to conventional treatment methods. Nevertheless, systemic administration of many of these therapies has also led to dose-limiting side effects (i.e., irAEs), highlighting the ongoing need for further improvements.^13^, ^14^, ^15^ A major goal of current research is to reduce drug-associated toxicities by directing immune-modulating therapies to the tumor site using multivalent, multispecific biologics. Below, we briefly review some of the monovalent therapies used in immunomodulation and how they have been adapted for multivalent use. Many of these reagents look at overcoming the immunosuppressive escape mechanisms mentioned above, either by blocking co-inhibitory signals, promoting co-stimulation, or altering the tumor microenvironment (TME) through neoantigen presentation and cytokine production. Tables 4 and 5 summarize several of the best-characterized monovalent and multivalent immunomodulatory aptamers, respectively. Immunomodulating antibodies have already been extensively reviewed and are cited in their respective sections below.

**Table 4:** Monovalent aptamers used in immunomodulation

Target (aptamer name)	Host	Nature	Reference
Agonist			

Immune Receptors			
4-1BB (*M12-23*^a^)	Murine	2′FY RNA	McNamara et al.^36^
OX40 (*OX40-apt9.8*^a^, *9C7T*^a^)	Murine and Human	2′FY RNA	Dollins et al.^37^, Pratico et al.^38^
CD28 (*CD28Apt7*^a^, *CD28Apt2*^a^)	Murine	2′FY RNA	Pastor et al.^39^
CD16a (*CLN0020*)	Human	DNA	Boltz et al.^40^
DEC205 (*min.2*^a^)	Murine	2′FY RNA	Wengerter et al.^41^
CD40 (*CD40Apt1*^a^)	Murine	2′FY RNA	Soldevilla et al.^42^
CD3ε (*OSJ-D-8S*^a^ derived from *ZUCH-1*)	Human	DNA	Freage et al.^43^

Antagonist			

Immune Checkpoints			
PD-1 (*A6*, *PD4S*, *MP7*, *XA-PD1-78*)	Murine and Human	DNA, X-aptamer	Gao et al.^44^, Prodeus et al.^45^, Wang et al.^46^, Li et al.^47^
PD-L1 (*B10*, *PL1*, *MJ5C*, *aptPD-L1*, *XA-PDL1-82*)	Murine and Human	DNA, X-aptamer	Wang et al.^46^, Li et al.^47^, Gao et al^48^, Lai et al.^49^, Huang et al.^50^
CTLA-4 (*M9-9/Del60*)	Murine	2′FY RNA	Santulli-Marotto et al.^51^
CTLA-4 (*aptCTLA-4*)	Murine and Human	DNA	Huang et al.^52^
TIM3 (*TIM-3 aptamer*^a^, *TIM3-Apt1*)	Murine	2′FY RNA	Gefen, et al.^53^, Hervas-Stubbs et al.^54^
LAG3 (*LAG3 Apt1*)	Murine	2′FY RNA	Soldevilla et al.^55^
Cytokines or Cytokine Receptors			
C5a (*AON-D21*^b^)	Murine and Human	RNA	Ajona et al.^56^, Hoehlig et al.^57^
CXCL2 (*NOX-A12*^b^)	Murine and Human	RNA	Klussmann et al.^58^, Vater et al.^59^
CCL2 (*NOX-E36*^b^)	Murine and Human	RNA	Kulkarni et al.^60^
IL6 (various SOMAmers)	Human	DNA	Gupta et al.^61^
TNFa (*VR11*)	Human	DNA	Orava et al.^62^
IL10R (*R5A1*)	Murine and Human	RNA	Berezhnoy et al.^63^, Levay et al.^64^
IL4Rα (*CL-42*)	Murine and Human	2′FY RNA	Roth et al.^65^
Immune Receptors			
BAFF-R (*R-1*)	Human	2′FY RNA	Zhou et al.^66^
CD28 (*CD28Apt2*)	Murine	2′FY RNA	Pastor et al.^39^
CD40 (*CD40Apt3*)	Murine	2′FY RNA	Soldevilla et al.^42^
Transcription Factor			
NF-κB (*A-P50*)	Human	RNA	Cassiday et al.^67^, Cassiday et al.^68^, Mi et al.^69^, Mi et al.^70^

Other/Unspecified			

IL6R (*AIR-3*)	Human	RNA	Meyer et al.^71^
4-1BB	Murine and Human	RNA	Levay et al.^64^
CD3ε (*OSJ, ZUCH-1, JZP O -10*)	Human	DNA	Freage et al.^43^, Zumrut et al.^72^, Zumrut et al.^73^

There are various other aptamers with affinity for solid tumor cell markers reviewed in Morita et al.^74^ NF, nuclear factor.

a Multimeric.

b L-aptamer (Spiegelmer).

**Table 5:** Multivalent aptamers used in immunomodulation

Tumor target (aptamer name) [nature]	Immune target (aptamer name) [nature]	Immune action	Linker	Reference
*1* + *1*				

c-Met (*CLN0003*) [DNA]	CD16α/FcγRIII (*CLN0020*^a^) [DNA]	NK cell and γδ T cell activation	Various ss linkers (15 deoxyA, 7-nt original untruncated anti-CD16 aptamer sequence)	Boltz et al.^40^
CD62L (*LD201t1*) [DNA]	Ramos cells (*TE02*); PTK7 (*sgc8*); PD-L1 (*aptPD-L1*) [DNA]	None	13-nt adapter (complementary) sequence and 2-nt ligated with T4 DNA ligase	Yang et al.^75^
Hepatocellular carcinoma (*TSL11a*) [DNA]	CD16 (*CLN0020*^a^) [DNA]	NK cell activation	Rigid Y-type DNA scaffold	Zheng et al.^76^,^b^

*1* + *2*				

VEGF (*ARC245*) [DNA]	4-1BB (*M12-23*) [2′FY RNA]	Co-stimulation	24-nt adapter (complementary) sequence extended on 3′ end of each aptamer	Schrand et al.^77^
OPN (*OPN-R3*) [DNA]	4-1BB (*M12-23*) [2′FY RNA]	Co-stimulation	24-nt adapter (complementary) sequence extended on 3′ end of each aptamer	Schrand et al.^77^
OPN (*OPN-R3*) [2′OMe RNA]	4-1BB (*M12-23*) [2′FY RNA]	Co-stimulation	18-nt adapter (complementary) sequence	Wei et al.^78^
PSMA (*A10*) [2′FY RNA]	4-1BB (*M12-23*) [2′FY RNA]	Co-stimulation	3′ end of PSMA aptamer had 21-nt complementary sequence to 4-1BB dimer	Pastor et al.^79^
MRP1 (*MRP1Apt*) [2′FY RNA]	CD28 (*CD28Apt7*) [2′FY RNA]	Co-stimulation	(2) CD28 and (1) MRP1 aptamer made from single PCR product with 19-nt linker between the two	Soldevilla et al.^80^

*2* + *2*				

MUC1 (*MA3*) [DNA]	CD16α/FcγRIII (*CLN0020*^a^) [DNA]	NK cell and γδ T cell activation	Four aptamers (two MUC1, two CD16) linked by (3) 60 nt A/C rich ss spacers	Li et al.^81^

Other				

CD62L (*LD201t1*) [DNA]	Ramos cells (*TE02*) [DNA]	None	Various dsDNA nanoscaffolds	Liu et al.^82^

ss, single stranded; ds, double stranded; deoxyA, deoxyadenosine.

a truncated version.

b not expanded upon in this review.

### Monospecific or monovalent therapy

Immunomodulatory antibodies and aptamers can be broadly categorized according to their mechanism of action as being either antagonists or agonists. Often these reagents are targeted to co-inhibitory or co-stimulatory immune receptors, respectively, but some have also been targeted to other immune receptors, cytokines, and transcription factors.

#### Monospecific antibodies

##### Antagonists of co-inhibition

In 1986, the US Food and Drug Administration (FDA) approved the first mAb, OK3 (muromonab), for therapeutic use in prevention of kidney transplant rejection.^83^ As of May 2021, 100 therapeutic mAbs have FDA approval, and some have become the predominant treatment modality for their corresponding diseases.^8^ Over the last decade, the FDA has approved various ICIs that display profound effects on inhibiting the progression of malignant tumors. Such approvals have accelerated since Dr. James Allison and Tasuku Honjo were awarded the Nobel Prize in Physiology or Medicine for the discovery of programmed cell death protein-1 (PD-1) and cytotoxic T lymphocyte-associated antigen-4 (CTLA-4) in 2018.

CTLA-4 is a lymphocyte predominant cell surface receptor. When bound by a cell surface anchored ligand such as CD80 (B7-1) or CD86 (B7-2), it produces a net inhibitory signal, preventing T cell activation. PD-1 (CD279) is also a cell surface receptor and is a member of the CD28 superfamily. When PD-1 is bound by its cell surface anchored ligands, PD-L1 (B7-H1) or PD-L2 (B7-DC), it too suppresses the activation and function of T cells. Both of these co-inhibitory receptors, as well as many others, are highly expressed in unresponsive, exhausted T cells.^84^ Thus, the various ICI antibodies function to prevent the inhibitory signal, pushing T cells in the direction of activation and pro-inflammatory cytokine production (e.g., interferon [IFN] γ and TNFα). Additionally, some of the ICIs have been shown to deplete tumor Treg cell populations, either by inducing antibody-dependent cell-mediated cytotoxicity (ADCC) or by downregulating forkhead box P3 (FOXP3), a protein that functions as a master regulator in the development and function of Tregs.^85^ There are currently seven FDA-approved ICIs against the targets CTLA-4 (ipilimumab), PD-1 (pembrolizumab, nivolumab, cemiplimab), and PD-L1 (atezolizumab, avelumab, durvalumab).^86^^,^^87^ Unique to a few of these therapies is that their FDA approval was based on biological oncologic mechanism regardless of underlying tumor type; thus, they can be used for a wide variety of cancer types. Despite the wide applicability of ICIs, the benefits are often restricted to patients with a TME that has an “inflammatory profile” and high PD-L1 expression. That is, most patients who respond to ICI therapy^88^^,^^89^ have a TME containing an increased number of tumor infiltrating lymphocytes (TILs) with a high T effector cell (Teff) to Treg ratio, high levels of neoantigen expression and mutational load, increased secretion of IFNγ and other pro-inflammatory cytokines, and low levels of MDSCs.^88^^,^^90^ Furthermore, many patients will acquire resistance to these ICIs, either through decreased target ligand expression or upregulation of other immunosuppressive genes such as T cell immunoglobulin and mucin domain containing 3 (TIM3).^85^ Beyond PD-1/PD-L1 and CTLA-4, there has been a push to find the next generation of ICIs to overcome resistance. There are now multiple mAbs in the pre-clinical and clinical pipeline that target novel, co-inhibiting immune checkpoint targets such as TIM3, lymphocyte activation gene 3 (LAG-3; CD223), T cell immunoglobulin and ITIM domain (TIGIT), V-domain immunoglobulin suppressor of T cell activation (VISTA), B7 homolog 3 protein (B7-H3; CD276), B and T lymphocyte attenuator (BTLA; CD272), and CD73.^91^^,^^92^ Their application in the co-blockade of multiple immune checkpoints has already shown promising results.^85^

##### Agonists of co-stimulation

Two mAbs, urelumab and utomilumab, have been developed to agonize CD137 (4-1BB/TNFRSF9), a member of the TNF receptor superfamily (TNFRSF). The TNFRSF consist of 29 receptors, classified into two groups: death receptors and activating receptors.^84^^,^^93^ 4-1BB acts as an activating receptor, primarily in the co-stimulation of T cells, for which interactions between T cell receptor (TCR) and MHC are still required for activation. Agonistic antibodies targeted to CD137 (anti-CD137) have been shown to improve anti-tumor immunity in several pre-clinical models of cancer.^94^ Both antibodies are currently being investigated in the clinical pipeline for various hematologic malignancies and solid tumors.^95^ Closely related, Hoffman and colleagues^96^ developed a monovalent anti-CD3ε Fab (Mono-7D6-Fab, derived from mAb 7D6) that co-potentiates T cell activation. The binding of mono-7D6-Fab to CD3 induces a conformational change in the CD3 complex (CD3Δc) that drives T cell activation in the presence of weakly binding antigens that would not normally activate T cells. Additionally, Fab binding to CD3 does not stimulate TCR signaling in the absence of antigen, and co-potentiation was dependent on antigen specificity, which greatly mitigates non-specific immune cell activation. The monovalent anti-CD3ε Fab has shown promising results in both *in vitro* and *in vivo* pre-clinical models, including reducing tumor burden in a murine model of melanoma lung metastasis.^96^ However, this reagent has not yet been described in clinical applications.

#### Monospecific aptamers

Aptamers with affinity for various co-inhibitory immune checkpoint targets have been selected, including all three traditional targets (CTLA-4, PD-L1, PD-1) and a few next-generation targets (TIM-3 and LAG-3). There have also been a few aptamers selected with affinity for co-stimulatory TNFRSF targets, including 4-1BB, OX40, and CD40. Unique to the aptamers for 4-1BB and OX40 is that, while their monomeric forms have no agonistic or antagonistic effect on their targets (i.e., silent binders), they become agonists when multimerized.^36^, ^37^, ^38^ The dependence of the outcome upon the multimerization state of the aptamer is consistent with the known requirement that signaling cascades downstream of these cell surface receptors are triggered upon crosslinking. In the case of co-stimulating receptors, this crosslinking must still be in the presence of MHC I-dependent antigen presentation to induce T cell activation. However, these data also establish that binding to the cognate ligand is not required for triggering cellular responses. These dimeric, monospecific aptamers were shown to activate cytotoxic T lymphocytes (CTLs; as measured by increased IFNγ release), prevent tumor growth *in vivo*, and prolong animal survival in various murine models.^36^^,^^37^ This example confirms that linking together monovalent aptamers could lead to a gain of new functionalities that are not displayed by mixtures of the parental molecules. An implication for rational engineering of multispecific/multivalent aptamers is that careful *in vitro* and *in vivo* evaluation is needed to establish the mechanistic basis of any potential new functionality or biological outcome, such as activation of certain signaling cascades. Two other notable immunomodulatory aptamers are the anti-CD28 aptamers (named *CD28Apt2* and *CD28Apt7*) and the anti-C5a Spiegelmer (L-aptamer; *AON-D21*). The anti-CD28 aptamers function as antagonists of the co-stimulatory receptor in their monomeric forms (demonstrated for *CD28Apt2*) and as agonists in their dimeric forms (demonstrated for *CD28Apt2* and *CD28Apt7*). The anti-CD28 dimer promoted T cell activation, induced strong cellular and humoral responses, potentiated vaccination, and improved animal survival in an *in vivo* lymphoma model.^39^ Spiegelmer AON-D21 showed synergistic anti-tumor effects when used in conjunction with an anti-PD-1 mAb, owing to lower MDSC populations and increased CD8^+^ T cells with decreased expression of exhaustion markers. This example reinforces the role of combined therapies to aid ICIs in reprograming the TME.^56^ Despite these promising discoveries, only two immunomodulating aptamers, *NOX E36* and *NOX A12* (antagonists of the chemokines CCL2 and CXCL2, respectively), have moved into the clinical pipeline for cancer treatment thus far.^29^^,^^97^^,^^98^ A comprehensive list of published immunomodulating aptamers is provided in Table 4, the majority of which have been extensively reviewed elsewhere.^26^^,^^74^^,^^97^^,^^99^^,^^100^

### Multispecific or multivalent therapy

Multivalent therapy offers the advantage of targeting an immunomodulatory signal directly to a cell population of interest. In practice, one part (or module) of the multivalent construct binds to an antigen on a tumor cell surface or in the TME and the other part binds to a distinct immune cell surface receptor, effectively bringing in close proximity these two cell types and creating an induced cell-cell junction. This close apposition, in conjunction with a specific immunomodulating function of the biologic, is often enough to elicit a powerful and targeted immune-mediated cytotoxic response. Importantly, designs of existing and effective bsAbs can help guide the rational engineering of bsApts with improved immunomodulatory properties (see section “improving immunomodulation” for further insights and details).

#### Bispecific antibodies

The majority of clinically approved T cell-engaging bsAbs (often referred to as bispecific T cell engagers [BiTEs]) target a TAA on the tumor cell surface and CD3, the invariant component of the TCR complex, on the CTL surface. Simultaneous binding of TAA and CD3 creates a cell-cell junction that serves as a functional “immune synapse” by inducing T cell activation and ultimately tumor cell lysis. The major benefit of bsAbs (and CAR T cells) is that they bypass the need for neoantigen presentation on MHC molecules, whereas ICIs are inherently reliant on MHC presentation. However, co-receptor signaling appears to still play an important role in bsAb efficacy.^101^^,^^102^ Blinatumomab, which targets CD19 and CD3, was the first FDA-approved BiTE and is now being used to treat various types of refractory and relapsed B cell acute lymphocytic lymphoma (R/R B-ALL), minimal residual disease (MRD^+^) B-ALL, and R/R diffuse large B cell lymphoma (R/R DLBCL).^103^ Since the proof-of-concept introduction of blinatumomab, various other BiTEs against hematologic and solid tumor targets (e.g., PMSA, EpCAM, EGFRvIII, DLL3, MUC17, CLDN18.2) have shown promising results in both pre-clinical and clinical studies. These and other novel bsAb constructs (e.g., DART, TandAb, CrossMAb, CiTE, SMITE, TriKE) are reviewed elsewhere.^103^, ^104^, ^105^, ^106^, ^107^ Knowledge obtained from bsAbs can help guide the way we engineer, categorize, and utilize bsApt constructs.

#### Bispecific aptamers

Immunomodulating bsApts can be categorized by both the valency and specificity of the two aptamer modules using an [*m* + *n*] nomenclature (Figure 2), where [*m*] corresponds to the valency of the tumor-targeting aptamer module and [*n*] corresponds to the valency of the immune cell-targeting aptamer module. For example, a [*1* + *2*] designation describes a construct composed of one aptamer that binds targeted tumor cells and two aptamers that bind immune cells. Most immune cell-targeting aptamers that agonize co-stimulatory receptors (e.g., CD28, 4-1BB, or OX40) indeed require a bivalent design ([*n*] = 2), because receptor crosslinking by their dimeric form is needed to induce signaling and T cell activation (as discussed above). Other aptamers that exert their effects as monomers (e.g., localization to TAA, natural killer [NK] cell activation, antagonization) can use a [*1* + *1*] design. Some bsApts with increased valency have been built with the primary goal of increasing avidity for their target, either on the tumor or immune cell (e.g., [*2* + *2*] or [*4* + *4*]). Although there are currently no trispecific immunomodulatory aptamers in the literature, their design can still be categorized using this nomenclature (e.g., [(*1* + *1*) + *1*]; see Figure 2). Apart from the number of the aptamer modules in each bsApt construct, other variables that have been explored, albeit superficially, are properties of the linker domain that connects the two aptamer modules, such as its length, composition, and rigidity.

##### [*1* + *1*] valency

**Figure 3:**
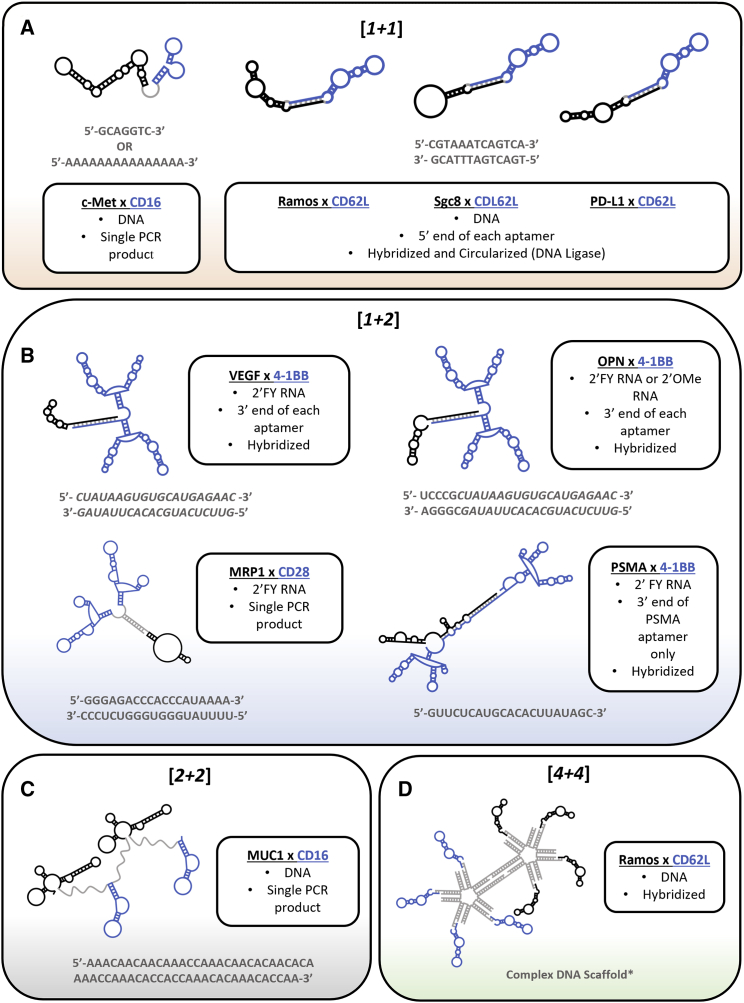
Immunomodulating bsApts Published immunomodulating bsApts covered in this review. Immune-cell-targeting aptamers (blue) and tumor-targeting aptamers (black). Boxes include target names (bold) and aptamer and linker features such as composition (e.g., DNA versus RNA) and synthesis method (e.g., hybridization versus single PCR product). If hybridized, method of linker extension is noted in the box (e.g., 3′ end of each aptamer). Circularized aptamers were hybridized and then ligated using DNA ligase. Linker sequences provided in gray below boxes. Conserved nucleotide sequences between linkers are in italics.

Boltz et al.^40^ selected multiple DNA aptamers with affinity for c-Met (HGF-R) and CD16a (FcγRIIIa). CD16a is an intermediate affinity Fc receptor found mainly on NK cells and to a lesser extent on γδ T cells, monocytes, and macrophages.^108^ This receptor is important for ADCC, phagocytosis, and clearance of immune complexes. C-Met is a multidomain receptor tyrosine kinase (RTK) expressed on cells of epithelial origin and is important for wound healing and embryonic development. Aberrant signaling of c-Met has been implicated in various cancers, including lung, colorectal, liver, and gastric cancers.^109^ Boltz and colleagues showed that two c-Met/CD16a bsApts (called *bsA17* and *bsA22*; Figure 3A) induced ADCC-mediated cell lysis with similar magnitude to an Ab control (anti-EGFR, cetuximab) when gastric and lung carcinoma cells were co-cultured with peripheral blood mononuclear cells (PBMCs). Interestingly, this group noted that linker lengths of ∼49–152 Å (equivalent to ∼7–22 nt) mediated significant cytotoxic effects, while linker length greater than ∼200 Å (equivalent to ∼29 nt) did not. Importantly, the distances found in the c-Met/CD16a bsApt constructs that induced cytotoxicity are similar to the immune synapse created by various bsAbs (∼100 Å) and to the physiological immune synapse created by TCR-peptide MHC (pMHC) binding (∼150 Å).^110^^,^^111^ The evidence presented by Boltz et al. suggests that linker length may play a significant role in the formation of a functional immune synapse, as further detailed below (see section “insights for new technological developments”).

**Figure 4:**
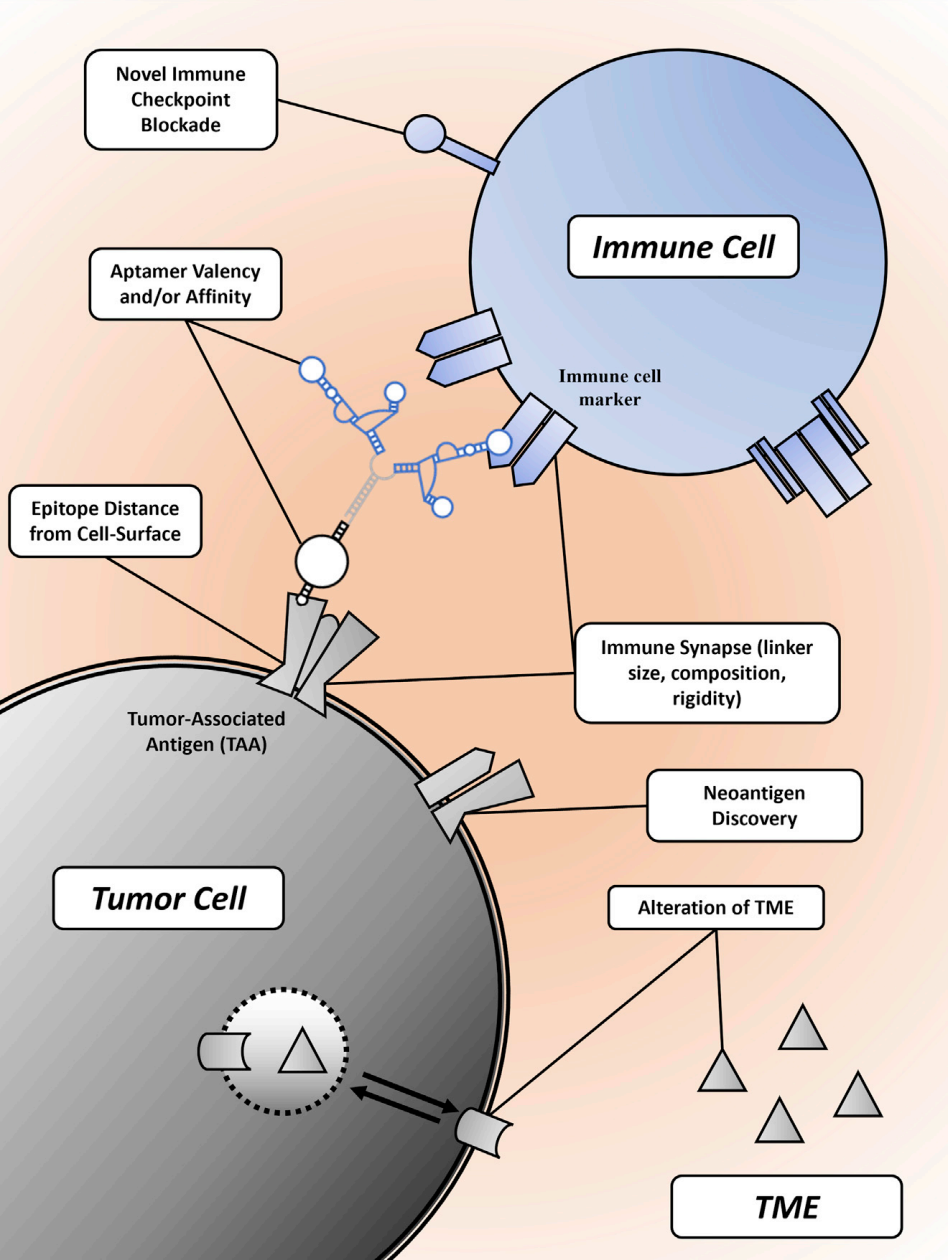
Considerations during bsApt development Highlights important factors to consider during the bsApt development process. This includes selection and post-selection molecular engineering. Relevant populations to consider include the tumor cell, immune cell, and TME.

Yang et al.^75^ conjugated a DNA aptamer with affinity for CD62L (aptamer *LD201t1*), which is present on naive T cells, to other aptamers that recognize PTK7 (aptamer *sgc8*), PD-L1 (aptamer *PD-L1*), or whole Ramos cells (B cell lymphoma; aptamer *TE02*). These aptamer conjugates were referred to as circular bsApts (c-bsApts; Figure 3A), as the 5′ and-3′ ends were ligated together using T4 DNA ligase after hybridization of a 13-nt flanking complementary sequence. Yang and colleagues reasoned that, without free ends, c-bsApts would be more resistant to degradation by serum exonucleases, consistent with previous observations.^112^^,^^113^ Indeed, while monovalent aptamer was almost fully degraded by 12 h in 10% fetal bovine serum (FBS), c-bsApts were still visually detectable by denaturing PAGE gel at 36 h. However, the magnitude of the protection afforded by circularization is not immediately apparent, as this study did not compare the *in vitro* half-life of each c-bsApt with its non-circularized counterpart (i.e., obtained upon hybridization of the two aptamers). It is also not clear that similar protection would be observed at higher concentrations of FBS that more closely mimic physiological conditions. Nevertheless, *LD201t1/TE02* c-bsApt (Figure 3A) induced artificial, *in vitro* cell-cell junctions of Ramos cells with Jurkat (T cells) and with C57BL/6 mouse extracted splenocytes. Interestingly, these cell-cell junctions were silent and did not induce T cell-mediated cytotoxicity of the targeted cell. Instead, cytotoxicity had to be induced by the addition of T cell activator beads coated with anti-CD3 and anti-CD28 mAb (Dynabeads). The group termed this phenomenon the recognition-then-activation model, which is in some respects conceptually similar to the co-potentiation model described by Hoffmann and colleagues.^96^ Cell-cell junctions were also observed with the *LD201t1/sgc8* c-bsApt (Figure 3A), which targeted splenocytes to PTK7 on CCRF-CEM cells. To evaluate the *in vivo* therapeutic potential of the recognition-then-activation model, a *LD201t1/PD-L1* c-bsApt (Figure 3A) was also tested in a B16-F10 (murine melanoma) tumor model, where combination of this c-bsApt with anti-CD-28 antibody-coated T cell activator beads showed decreased tumor growth and increased survival compared with controls. The group also noted that mice treated with this recognition-then-activation model had strong immune memory, shown by increased Teff cell populations (CD3+/CD8+/CD44+/CD62L-) at 44 days post treatment compared with mice that received surgery alone.

The [*1* + *1*] designs are effective at bringing immune cells in close proximity to tumor cells to create either a silent cell-cell junction or a cytotoxic immune synapse, as indicated by multiple measures. The evidence presented by Boltz et al. suggests that the creation of a cytotoxic immune synapse is highly dependent on linker length. We note that, to date, no [*1* + *1*] designs have been constructed using antagonistic aptamers such as those targeting co-inhibitory immune checkpoints. We speculate that such a design could provide additional opportunities to improve therapeutic indices and potency of such reagents by targeting immune checkpoint blockade to cancer cells and by inducing T cell activation from induction of artificial immune synapses, respectively.

##### [*1* + *2*] valency

The [*1* + *2*] design increases the valency of the aptamer that targets the immune cell. To date, all immunomodulating bsApts described using this design contain a dimeric aptamer that agonizes a co-stimulatory receptor on the immune cell. Pastor et al.^79^ hybridized their previously selected agonistic, dimeric 2′FY RNA aptamer (*M12-23*) that binds 4-1BB to a 2′FY RNA aptamer (*A10*) that targets prostate-specific membrane antigen (PSMA). PSMA is a cell surface receptor that is upregulated in human prostate cancer.^114^ The *M12-23* aptamer was selective for human PSMA (hPSMA) and does not recognize murine PSMA (mPSMA). Murine CT26 colorectal cancer and B16-F10 tumor cells were stably transfected with an hPSMA expression plasmid to serve as surrogates for human prostate cancer. Additionally, to simulate a non-internalizing receptor, 13 amino acids were deleted from the cytoplasmic domain of PSMA (ΔPSMA), which reduced receptor internalization upon aptamer or ligand binding.^115^ This design favored retention of the anti-hPSMA/anti-4-1BB bsApt on the tumor cell surfaces, which in turn provided a potential window of opportunity to recruit immune cells and form an induced synapse. Indeed, the anti-hPSMA/anti-4-1BB bsApt ([*1* + *2*]; Figure 3B) was able to inhibit tumor growth and increase survival in a subcutaneous colorectal cancer model, as well as prevent lung metastasis in mice implanted with ΔPSMA-B16-F10 cells. The anti-hPSMA/anti-4-1BB bsApt also potentiated vaccination (GVAX) to prevent lung metastasis. Pastor and colleagues showed that the anti-tumor effect was seen at a 10-fold lower dose (50 pmol versus 500 pmol) for the hPSMA/4-1BB bsApt compared with either a monovalent aptamer or mAb against 4-1BB. This anti-cancer effect was also dependent on the expression levels of ΔPSMA on the tumor cell surfaces. In addition, hPSMA/4-1BB bsApt displayed reduced off-target (off-tumor) immune effects compared with anti-4-1BB mAb, as measured by decreased spleen, lymph node, and liver mass and lower CD8^+^ T cell populations (%). Similarly low levels of immunotoxicity in non-tumor tissue were seen with anti-4-1BB aptamer relative to mAb, suggesting a superior safety profile for aptamers, either as bsApts or monospecific reagents, over Abs.

The work of Pastor and colleagues^79^ was expanded upon when Schrand et al.^77^ conjugated the anti-4-1BB dimeric aptamer with previously selected DNA aptamers with affinity for vascular endothelial growth factor (VEGF) or osteopontin (OPN). VEGF and OPN are both secreted into the stroma by many tumors of different origins. The rationale behind targeting these proteins was that (1) they could enable targeting of diverse tumor types, and (2) the proteins were not rapidly turned over or internalized upon ligand/reagent binding or via constitutive endocytosis, as is the case for PSMA, EGFR, Her2, and transferrin receptor (TfR).^116^, ^117^, ^118^, ^119^, ^120^, ^121^ The anti-VEGF/anti-4-1BB bsApt ([*1* + *2*]; Figure 3B) showed anti-tumor effects at a dose five times lower than monovalent aptamer or mAb against 4-1BB (150 pmol versus 800 pmol). Furthermore, compared with monospecific reagents, VEGF/4-1BB bsApt decreased tumor size and increased survival in multiple tumor models, including breast cancer and two autochthonous models of fibrosarcoma and high-grade glioma. They also showed that the bsApt potentiated GVAX in a breast cancer postsurgical metastasis model, as well as in a melanoma model. Both Schrand and Pastor noted a two to five times increase in bsApt accumulation at the tumor expressing their protein of interest (VEGF and ΔPSMA, respectively), compared with the non-expressing tumor, as measured by accumulation of radioactively (^32^P) labeled 4-1BB aptamer.

Anti-OPN aptamer (2′OMe pyrimidine modified) and anti-4-1BB dimeric aptamer were linked together to form an OPN/4-1BB bsApt (1 + 2; Figure 3B).^78^ OPN is a glycophosphoprotein that is expressed intra- and extracellularly by various cell types. OPN is overexpressed in glioblastoma (GBM), one of the most malignant cancers, with only a poor (5%) 5-year survival rate despite aggressive treatment. OPN levels correlate with glioma grade, angiogenesis, and poor prognosis.^78^ OPN has three main roles in tumor progression: (1) it aids the TME by promoting MDSC expansion; (2) it suppresses anti-tumor immunity by promoting extramedullary myelopoiesis; and (3) it increases angiogenesis and tumor growth by upregulating COX-2 expression in tumor macrophages.^122^ Both monovalent aptamer and mAb against OPN were capable of blocking M0 and M2 macrophage migration, but they also both lacked a therapeutic effect *in vivo*. In contrast, OPN/4-1BB bsApt showed increased survival in the murine model of GBM. The authors noted increased CD3+ T cell populations and accumulation of the anti-4-1BB aptamer (via *in situ* hybridization using a 4-1BB antisense probe) in the intracerebral tumor when using the OPN/4-1BB bsApt, which was not evident with monovalent aptamer therapy. This suggested that the dual specificity was responsible for both T cell localization and increased cell survival.

Soldevilla et al.^80^ selected a 2′FY RNA aptamer with affinity for the extracellular domain of multi-drug resistance protein 1 (MRP1). MRP1 is a transmembrane protein that is highly expressed on cancer stem cells and has also been implicated in long-term chemotherapy resistance.^123^, ^124^, ^125^ The group used a phycoerythrin-labeled anti-MRP1 aptamer (PE-*MRP1Apt*) to isolate (sort) and enrich a subpopulation of B16-F10 melanoma cells with high levels of MRP1 that were genotypically and phenotypically resistant to chemotherapy. The group then developed an anti-MRP1/anti-CD28 bsApt ([*1* + *2*]; Figure 3B) by linking MRP1Apt to their previously selected, CD28 targeting 2′FY RNA dimeric aptamer (*CD28Apt7*). As expected, MRP1/CD28 bsApt showed a significant increase in the activation and proliferation of T cells (mediated by CD28 co-stimulation) *in vitro* and *in vivo* compared with monovalent aptamers. MRP1/CD28 bsApt showed increased accumulation at MRP1+ melanoma cancer cells (enriched population) compared with the parental melanoma cells, as measured by RT-PCR of disaggregated tumor. This bsApt also induced an immune response (infiltration of CD3+ lymphocytes at tumor with increased expression of IFNγ, TNFα, and IL-10) and potentiated a combination of GVAX and transient Foxp3 blockade via the P60 peptide (resulting in decreased tumor size and increase survival) *in vivo*. The group also created a novel tumor vaccine, called CD28Aptavax, in which they irradiated B16-MRP1 cells and decorated them with the bsApt. This novel vaccination method, but not control vaccination (irradiated B16-MRP1 cells decorated with unconjugated aptamers), elicited a strong immune response (increased T cell proliferation and IFNγ production) and inhibited subsequent tumor growth *in vivo*.

The [*1* + *2*] designs above were able to induce formation of a cytotoxic immune synapse. Importantly, all studies using this design included *in vivo* applications that showed increased animal survival and/or decreased tumor growth in various murine models of cancer. We note that, to date, no [*2* + *1*] designs have been described, nor have any [*1* + *2*] designs that do not contain an agonistic, co-stimulating dimer (e.g., anti-4-1BB aptamer). We speculate that [*2* + *1*] or [*1* + *2*] designs could be used to increase avidity for the tumor cell or immune cell, respectively, and improve synapse formation. Additionally, a [*2* + *1*] design could employ an auxiliary method of tumor cell lysis such as modulation of cell surface receptor signaling to induce apoptosis (expanded upon in the section “improving tumor recognition”).

##### [*2* + *2*] valency

BsApt designs in which both aptamers are dimerized could enhance immunomodulation by increasing avidity for both cancer cells and immunological cells, although this architecture has received relatively little attention. A tetravalent, bispecific [*2* + *2*] aptamer was recently constructed that consisted of two anti-MUC1 DNA aptamers and two anti-CD16a DNA aptamers.^81^ MUC1 is a transmembrane protein that is typically expressed in glandular and luminal epithelial cells of various tissues. This protein is overexpressed (and often aberrantly glycosylated) in almost all adenocarcinomas, and it is a marker of tumor initiation, progression, and prognosis.^81^^,^^126^ Anti-MUC1 aptamer, previously selected by the same group, and anti-CD16a aptamer, selected by Boltz and colleagues, were linked together by three different single-stranded DNA (ssDNA) spacers, all of which were A/C rich and 60 nt in length. The group found that the anti-MUC1/anti-CD16a ([*2* + *2*]; Figure 3C) bsApt but not the control could recruit CD16-positive immunocytes (PBMCs) to MUC1-containing A549 cells. Furthermore, they found that anti-MUC1/anti-CD16a bsApt significantly increased immune cell-related cytotoxicity (mediated by ADCC) of A549 cells, but not MUC1-negative HepG2 cells, compared with monovalent aptamers. In addition to these promising results, this study claimed that their first-in-class, tetravalent, bsApt ([*2* + *2*]) strengthened binding to both tumor and lymphocytes. However, the negative control against which their 420 nt anti-c-Met/anti-CD16a bsApt was compared was a random sequence, 20-nt probe, and no data were provided comparing binding avidity of this [*2* + *2*] bsApt with those of a similar sized controls, nor with those of its lower-valency, bispecific counterparts ([*1* + *1*], [*1* + *2*], [*2* + *1*]).

##### Higher-order valencies

Liu et al.^82^ generated a branched DNA nanoscaffold to create a bsApt with a [*4* + *4*] valency pattern (hybridization of two tetrameric, monospecific constructs; the author refers to this as “bispecific hetero-octamer”). The design combines aptamers *TE02* with affinity for Ramos cells and *LD201t1* with affinity for CD62 on T cells. These are the same aptamers used by Yang and colleagues to create c-bsApts described above. The [*4* + *4*] bispecific hetero-octamer (Figure 3D) successfully induced cell-cell interactions of Jurkat and Ramos cells, with significantly more cell-cell junctions occurring when the modules were connected via a more rigid structure in which the two five-point-star tiles were held together by a 15-nt double-strand linker (37.2% of the cells forming junctions), instead of an 8-nt double-strand plus 5-nt single-strand linker (22.1%). They attributed this effect to the single-strand-containing linker being too flexible to form a stable junction (i.e., the linker was not thermodynamically stable in solution). The group also studied a nanoscaffold that contained a [*13* + *13*] valency pattern using the same aptamers. Interestingly, they saw no further increase, and even a slight decrease, in the cell-cell linkage efficiency relative to the [*4* + *4*] design. When this group compared the TE02 aptamer alone, in various valency states and rigidities (mono, di, tri, and tetra; rigid versus non-rigid), they found that the tetramer had the lowest half maximal inhibitory concentration (IC_50_), followed by rigid trimer and rigid dimer, followed by non-rigid structures. They also noted that higher-valency aptamers had improved stability against nuclease in serum, likely due to an increased steric hindrance that reduces aptamer accessibility to nucleases, and that this improved stability was their initial rationale behind creating the bispecific hetero-octamer.

The [*2* + *2*] and higher-order valency designs were successful at inducing a cytotoxic immune synapse and bringing immune cells in close proximity to tumor cells for the formation of a silent cell-cell junction, respectively. These designs were ultimately used to improve avidity to both the tumor and lymphocytes; however, no direct comparison was done between the [*2* + *2*] design and other bispecific designs (e.g., [*1* + *1*], [*1* + *2*], [*2* + *1*]). Contrary to expectations, Liu et al. showed that increasing valency from [*4* + *4*] to [*13* + *13*] did not increase the number of achieved cell-cell junctions, suggesting that increasing the valency may not always improve avidity. One possible explanation for this finding is the existence of an “avidity gain” cap.^127^ Once reached, further increase in aptamer valency does not drive further increase of target cell binding. This cap likely depends upon multiple factors, including bsApt design and the expression levels and spatial distribution of the target receptors. While the mechanistic basis behind the authors' observations remain unclear, we speculate that linker properties such as length and rigidity play important roles. Nonetheless, immunomodulating bsApts that employ a higher-order valency design are under-investigated, including their application *in vivo*, and thus more studies are warranted.

## Insights for new technological developments

### Aptamers possess unique properties that are comparable with or even advantageous over protein-derived reagents used for immunomodulation of cancer

Abs have been indispensable reagents for the treatment of cancer and other diseases. In addition, immunomodulatory ligands are showing promise as anti-cancer reagents.^128^^,^^129^ Aptamers, while not yet widely accepted for clinical use, have proved able to function at least as well as their protein-derived counterparts in pre-clinical studies (Table 2). With continued awareness and amplification of research efforts, aptamers could soon provide clinicians with an adjuvant or alternative to Ab therapy. Immunomodulating bsApts highlight some of these advantages. First, aptamers can be screened *in vitro* quickly and cost-effectively, and they can be rationally engineering to form bispecific constructs via simple covalent (e.g., conjugation, ligation, co-transcription) or non-covalent (e.g., hybridization) methods. Each construct can have tailored properties to improve target receptor recognition and immunomodulation. Unique to aptamer technology, the geometry, rigidity, and length of the linker domain can be programmed to have different physicochemical properties; for example, by joining the modules via a double-stranded oligonucleotide or a chemical linker such as polyethylene glycol (PEG). Chemical modification of aptamers during or after selection^32^^,^^34^ is generally straightforward and is often used to increase serum half-life and improve stability, biodistribution, and bioavailability (Table 3).^130^ Moreover, bsApts can be easily scaled up with minimal batch-to-batch variability. Unlike CAR T cells and Abs, the production chain for bsApts does not include complications associated with genetically modified organisms, cell lines, or animal models.^131^ In contrast, bsAbs generally require both labor- and cost-intensive animal models to produce (with the exception of phage display technology), and stringent quality control measures are required to prevent co-purification of unwanted biological contaminants.^132^ These constraints reduce scalability and increase the potential for batch variability. While a biochemical background is often required for production of both aptamers and Abs, a strong protein engineering background is also generally needed to engineer bsAbs or CAR T cells. This engineering process requires delicate tuning of the individual binding affinities to reduce interactions with cells that express only one of the two markers. Ultimately, it could be technically challenging, expensive, and time consuming.^107^^,^^133^ Furthermore, modification and re-engineering of Abs are only possible under limited circumstances, constrained by their production methods.^134^

As foreign materials, oligonucleotides and protein have significant differences in their interaction with the immune system. Perhaps one of the most salient characteristics of aptamers is a low immunogenicity that is comparable with or even advantageous over humanized mAbs.^135^ Multiple studies have concluded that, unlike Ab controls, both monovalent aptamers and bsApts do not illicit significant non-specific immune cell activation, as measured by liver, spleen, and lymph node masses and CD8^+^ T cell populations, which all tend to be similar to those of untreated or vehicle controls.^77^^,^^79^ Unfortunately, many studies have not explored other potential off-target or on-target/off-tumor effects of immunomodulating bsApts, and no study directly compares bsApts with their bispecific protein counterparts, BiTEs. In principle, targeting an immune response directly to tumor cells using bispecific biologics could prevent such effects. However, various bsAbs tested in clinical trials (including FDA-approved blinatumomab) still had significant side effects in a subset of patients, such as leukopenia, cytokine release storm (CRS), and immune effector cell-associated neurotoxicity syndrome (ICANS).^105^^,^^136^ These side effects underscore the importance of studying potential off-target, or on-target/off-tumor, effects in pre-clinical models. A few studies did, however, use xenograft control tumors that did not express the targeted tumor marker on the cell surface.^77^^,^^79^^,^^80^ These studies concluded that only tumors expressing the targeted marker exhibited decreased tumor growth and increased bsApt accumulation at the tumor site (as measured by RT-PCR or ^32^P labeling of disaggregated tumors). Additionally, the anti-tumor effects of these bsApts were seen at markedly lower doses (5–10 times lower) compared with mAb or monovalent aptamer alone. These observations of increased potency and minimal off-tumor accumulation suggest an increased safety profile (i.e., therapeutic index) for bsApts.

Another advantageous property of aptamers in immunomodulation is the absence of complications arising from the antibody constant region (Fc). Fc has been shown to limit the cytotoxic effector function of T cells (i.e., limit therapeutic effect) by inducing ADCC, during which Teff cells are removed from the TME, either though the degranulation of NK cells and/or through phagocytosis by macrophages and monocytes. The combination of Teff cell depletion, the subsequent release of cytokines (i.e., CRS), and complement C1q binding, all driven by Fc, have been huge limitations to many mAbs and to the bsAb, catumaxomab (CD3 x EpCAM), which was voluntarily removed from the US market in 2013 for commercial reasons.^107^^,^^137^ While Fc-mediated effector functions can be avoided by removing or mutating the Fc region, as is now done with many BiTEs, this obstacle is completely avoided with aptamers.^91^^,^^107^^,^^138^

A final advantageous property of aptamers in immunomodulation is the ability to employ antisense antidotes to rapidly reverse adverse events. Through a mechanism of base-pair nucleation, strand exchange, and branchpoint migration, these antidotes can disrupt the folded structure of the aptamer and reverse binding to its target, thereby preventing the progression of serious adverse events such as those mentioned above.^139^^,^^140^ Immunogenicity, manufacturability, pharmacokinetics, and convenience are just as important as potency when designing bispecific therapies.^104^ On these fronts, aptamers outcompete Abs in many respects.

### Half-life of monovalent aptamers, but not bsApts, has limited their therapeutic potential

Monovalent aptamers have a significantly lower circulating half-life (minutes to hours) compared with mAbs (weeks), typically attributed to aptamer degradation by serum nucleases and renal excretion due to their polyanionic nature and small size (∼1–3 nm; ∼10–25 kDa). In contrast, the half-lives of bsAbs and bsApts are similar (hours to days, depending on construct type and valency). The shorter half-lives of bsAbs relative to mAbs arises from two factors. First, these constructs are significantly smaller than mAbs (∼55versus ∼150 kDa). As the glomerular filtration cut off is ∼50–60 kDa, bsAbs are in part expected to be renally excreted similarly to aptamers, albeit at a slower rate.^140^, ^141^, ^142^ Second, these constructs lack Fc, which is required for Ab recycling by the neonatal Fc receptor (FcRn) and is responsible for maintaining immunoglobulin G and albumin in circulation via transcytosis across polarized cellular barriers.^106^^,^^143^ Some of the new bsAbs, such as half-life-extended (HLE) BiTEs, re-attach the constant region with the ultimate goal of restoring favorable pharmacokinetics.^103^ However, this approach risks impeding T cell effector function and inducing the cytokine and complement-driven side effects mentioned above (unless the Fc region is mutated). Analogous approaches could be taken to improve half-life in bsApts. Ultimately, a short half-life means that therapies must be continuously infused during the period of treatment, and this can limit efficacy for low-potency reagents that require high volumes to deliver a dose that exerts their therapeutic effect. However, a shorter half-life will enable rapid recovery from any toxic sequelae and less risk of serious adverse events, which make aptamers attractive reagents, as is, for *in vivo* imaging and diagnostics.

### Various types of linkers are used in bsApt constructs

The linker segments connecting the aptamer modules of a bsApt are critical design parameters for ultimately determining the fate and quality of the artificial immunologic synapse. All linkers for published bsApt described in this review are summarized in Table 5 and Figure 3. While the length, composition, and rigidity of the linker have emerged as playing especially important roles, evaluations of these variables are largely lacking and thus a systematic approach is warranted.

Most immunomodulating bsApts have used either ssDNA or double-stranded oligonucleotide linkers with a length of 15–25 nt (∼105–175 Å). Only one study looked at the effect of linker lengths on induction of an immune response. They found that linker lengths of 7–22 nt (∼49–152 Å) mediated significant cytotoxic effects, while linker length over 29 nt (∼200 Å) did not.^40^ The length dependence of these effects is consistent with the kinetic segregation model (see section “improving immunomodulation)”, as these distances are similar to the physiologic immune synapse created by TCR-pMHC binding (∼150 Å) and by various bsAbs (∼100 Å).^110^^,^^111^ While this study compared the cytotoxic effects of various linker lengths using various combinations of newly selected aptamers, the authors did not directly compare linker lengths using the same two aptamers; such a study may be warranted to draw rigorous conclusions.

Linkers can be composed of various biosynthetic structural units, such as nucleotides, peptides, organic chains, and inorganic material. For studies involving immunomodulating bsApts, the aptamer modules have most often been connected via oligonucleotide linkers. Different sequences were used in nearly every bsApt construct (Table 5 and Figure 3). The groups that used the agonist anti-4-1BB aptamer are the exception, as all of these studies used similar sequences but hybridized the aptamers at different locations (see section “bsApts”). Linker composition also plays a role in determining its rigidity, which can affect conformational entropy and 3D presentation of the binding domains. Most studies have utilized double-stranded linkers and thus constitute a more rigid design. Only two groups used less-rigid, single-stranded linkers.^40^^,^^81^ Those two groups, along with Soldevilla et al.,^80^ synthesized their bsApts as one long product. In other studies, the aptamer domains have been linked using base-pair hybridization of complementary sequences added to either the 5′ or 3′ aptamer ends. One group showed that a flexible linker containing both ssDNA and double-stranded DNA (dsDNA) portions (versus dsDNA only) was less efficacious at making cell-cell junctions, which they attribute to poor thermostability.^82^ Similar observations were noted by Miao and colleagues, who constructed a non-immunomodulating bsApt against IGFIIR (a cell surface lysosome shuttling receptor) and c-Met, both of which are tumor cell antigens, to induce receptor internalization and degradation. They showed that a bsApt construct containing dsDNA linker (*D3*) was more efficacious at inducing receptor internalization and was more stable in 10% FBS than constructs containing ssDNA (*D1*) or combining dsDNA and ssDNA linkers (*D2*). Half-lives in 10% FBS measured by native PAGE were 24 h (*D3*), 10 h (*D2*), and 1 h (*D1*).^144^ Note that *in vitro* resistance to serum nucleases might differ from those observed *in vivo*. In fact, composition and concentration of serum nucleases in commercial FBS (bovine) can differ from those found in murine and human sera.^145^ Therefore, to make *in vitro* assessments of nuclease resistance as informative as possible, future studies should be performed using high serum concentration (≥50%) and possibly testing different types of serum. A study by Zheng and colleagues explored the impact of linker rigidity in the context of a bsApt with affinity for two different tumor cell antigens (EpCAM and CD44). However, they showed that the two or three unpaired nucleotides that flanked their 23-nt adapter sequence were crucial for functioning. The bsApt lost both its specificity and cytotoxic effects when they used a fully paired linker or one with only one unpaired nucleotide. The authors suggested that these unpaired nucleotides provided the spatial freedom for each aptamer to form its 3D structure.^136^ Finally, a fourth group suggested that free 5′ or 3′ sticky ends made their bsApt more susceptible to nucleases (see section “multispecific or multivalent therapy”: [*1* + *1*]), although no data were presented to address this question directly.^75^ While some of the outcomes from these four studies may seem to provide opposing guidance, the differences in linker composition, linker length, and aptamer sequences or targets make interpretation difficult. Furthermore, no studies have systematically evaluated these three properties with respect to immune-mediated cytotoxicity and, thus, no specific composition or synthesis method has yet emerged as being superior to others.

The linkers used in each study suggest that a wide variety of properties may be acceptable for proof-of-concept designs as long as the linker does not affect aptamer folding and stability. To move past proof-of-concept designs, it will be important to understand the extent to which each of these linker properties determines the ability of the bsApt to elicit an efficient anti-tumor response. However, it may be inherently difficult to derive general rules for optimal linker design, because, as we have learned from bsAbs, optimal linker lengths may differ depending on the cell types, the antigens that are targeted, and distances of the targeted epitopes from the cell surface.^104^^,^^107^

### The importance of valency in immunomodulatory bsApts is understudied

Apart from agonistic, co-stimulating immune-cell-targeting aptamers (e.g., anti-4-1BB dimeric aptamer), which are well studied and require a valency of at least two to exert their effects,^36^^,^^37^^,^^39^^,^^41^^,^^42^ the importance of valency in bsApt constructs is otherwise understudied. While increasing valency of an aptamer has been shown to increase avidity for its receptor, the ultimate effect on immune-mediated cytotoxicity remains unknown.^82^^,^^146^ Various valency states have been used in published studies of immunomodulatory bsApts, but only two such studies have made claims about the importance of the valency of their constructs. Li et al.^81^ stated multiple times that their MUC1/CD16a ([*2* + *2*]) bsApt was able to bind more avidly and displayed an increased cytotoxicity compared with MUC1/CD16a ([*1* + *1*]) bsApt, but without presenting data to support these claims. In a different report, Liu et al.^82^ constructed the anti-Ramos cell/anti-CD62L ([*4* + *4*]) bsApt with affinity for malignant Ramos cells and CD62L. They showed that increasing the valency from [*4* + *4*] to [*13* + *13*] slightly reduced the number of artificial cell-cell junctions, rather than increasing that number. No cytotoxic effect was noted for either design, as neither bsApt directly elicited an immune function by itself. They did, however, show that increasing the valency of a single aptamer (*LD201t1*; not used in a bsApt construct) using a DNA nanoscaffold increased avidity for its target receptor (CD62L) and thereby decreased IC_50_ values.

The paucity of information regarding the effects of increasing valency of bsApts on immune-mediated tumor cytotoxicity warrants more studies comparing such constructs using the same aptamers with varying valency to delineate the underlying determinants of immunomodulatory outcomes. One possibility is that cytotoxicity is dependent on the tumor target of interest. For example, increasing aptamer valency toward an RTK (e.g., EGFR) might transiently increase apparent affinity for the targeted cells. However, this increase of avidity might also enhance affinity for non-malignant cells that still expressed the targeted RTK at lower levels, leading to undesired on-target/off-tumor effects. In addition, bringing two RTKs into close proximity can increase receptor internalization by endocytosis and reduce the net cytotoxicity induced by bsApts^147^^,^^148^ that aim to connect tumor and immune cells to create an artificial immune synapse. While triggering internalization may be a disadvantage for immune synapse formation, it could be beneficial for the delivery of a therapeutic cargo (e.g., small interfering RNA [siRNA] or chemotherapeutic) inside target cells. Therefore, aptamer valency should be tailored based on the ultimate biomedical application of that given bsApt construct. Fortunately, the ease of aptamer engineering offers the opportunity to generate and screen many different bsApts in a timely manner and at reduced cost, with each one displaying unique functionalities that address a given biomedical problem.

### A need for better control aptamers and cell lines

While most studies on bsApts have included one or more control aptamers and cell lines appropriate for attenuated interpretations of the findings, many lack the controls needed for robust interpretation of the underlying biology. Only a few studies included aptamer controls that were comparable with the experimental group, such as mutant or scrambled bsApts that do not bind the target receptors. Many studies also lacked appropriate negative control cell lines, such as those in which the purported target was knocked out, knocked down, or minimally expressed. Additionally, while many studies compare bsApts with traditional mAbs, bsApts should also be compared with bsAbs—which are mechanistically more analogous to bsApts—and with conventional therapies such as chemotherapeutics. It can be time consuming, labor intensive, and costly to include such controls alongside experimental groups. Nevertheless, the rigor provided by well-controlled studies is imperative to facilitate clinical translation of these aptamers into the clinical pipeline.

## Improving immunomodulation

### The immune synapse: Applying lessons learned from BiTEs

With the introduction of more than 80 bsAbs into the clinical pipeline for cancer therapy,^107^ there has been much interest in how to make these biologic reagents more efficacious. Even in the face of BiTE therapy, cancer cells can evade immune recognition through multiple mechanisms, including loss of tumor antigen, upregulation of immune checkpoint proteins, and formation of an immunosuppressive microenvironment. It is imperative that we take a deliberate approach in creating the next generation of bispecific therapies. Creation of an effective immune synapse appears to be crucial to enhancing cytotoxicity. Three factors stand out as being especially important for bsAbs: (1) CD3-mediated MHC I bypass, (2) physical distance spanned by the bispecific reagent, and (3) the number of synapses formed between the cancer cell and the immune cell. It is likely that these factors must also be considered when designing bsApts for immunomodulation.

The first component to consider when creating an effective immune synapse is the potential for bypassing or mitigating the need for MHC I-mediated TCR activation by targeting CD3. CD3, the invariable T cell co-receptor, is composed of four distinct extracellular subunits (two ε, one γ, and one δ). While BiTEs have targeted αβTCR, CD5, CD28, and CD2 with limited success, the most well-understood constructs target the non-glycosylated CD3 subunit epsilon (CD3ε). Upon binding of bsAbs to both TAA and CD3ε, T cells quickly become activated.^107^^,^^149^ This is generally attributed to the kinetic segregation model, which states that close apposition of the two cell membranes excludes the bulky extracellular domain of CD45, a protein tyrosine phosphatase. This exclusion permits the intracellular phosphorylation of TCR and associated proteins to be maintained, leading to sustained signal transduction and activation.^104^^,^^150^ This model, as well as other models of TCR activation (e.g., the mechanosensory model^151^^,^^152^), can help guide the production of new bsApt therapeutics, such as those targeted to block CD45 or to bind CD3ε.^43^

A second vital component to consider is cell-to-cell distance, which is determined largely by the antigens and epitopes being targeted and by the architecture of the bispecific construct. Cell membrane proximal epitopes force membranes close together, and bsAbs that target them are associated with more potent synapse and thus more T cell activation,^153^ consistent with the kinetic segregation model above. In contrast, distal epitopes on bulky antigens create larger, less potent synapses and thus less T cell activation.^104^ For bsApts, the linker type and length can be adjusted through appropriate molecular design and also contribute to cell-to-cell distance. With the average physiological immune synapse spanning ∼150 Å and the average bsAb immune synapse spanning ∼100 Å, we suggest that similar distances should be targeted in the construction of bsApts.^110^^,^^111^ The findings of Boltz et al.^40^ mentioned above support the claim that an overtly *large* synapse limits cytotoxicity. However, it has not been well established whether an immune synapse can be too *small*. Defining the boundaries of the immune synapse created by bsApts for maximal cytotoxic effects is thus warranted.

The third important factor is the number of individual synapses that are needed to induce the cytotoxic effects of immune cells. For the native TCR-pMHC complexes, as few as 10 synapses may be sufficient to induce activation,^107^^,^^154^ but this number likely depends on antigen strength.^96^ However, the number of synapses that need to be formed by bispecific biologics to induce activation is unknown and highly debated. The actual number likely depends upon properties intrinsic to the TAA as well as synapse characteristics such as distance, target affinity, and aptamer valency. The relative contributions of affinity and valency of bsAbs remain elusive, likely due to the lack of studies directly comparing similar antibody clones with different valences and affinities. Traditionally in pharmacodynamics, increasing affinity and avidity increases potency; therefore, more T cell activation is expected upon affinity maturation and increased valency. This may, however, not be in alignment with the “productive hit rate” model of T cell activation, which states that it is the *relative number* of TCR-pMHC interactions *of sufficient duration* (governed by off rate, k_*off*_), not the absolute affinity or avidity, that determines efficiency of activation.^104^^,^^155^, ^156^, ^157^ Thus, downstream cytotoxic effects, which are the measure of potency in this context, may not be driven primarily by affinity or avidity. In support of this, BiTEs have shown only weak correlations between TAA avidity and *in vitro* potency.^104^ One way to characterize the number of synapses needed to induce immune cell cytotoxicity is to explore how the number and spatial distribution of TAAs on a cell surface determine the reagent's effectiveness. Watanabe et al.^158^ showed that, for CAR T cells that recognize CD20, the minimum number of TAAs per cell was 1,000 to induce 50% lysis and >5,000 to produce maximal lysis. The corresponding data for bsApts are not yet available.

Ultimately, all three of these factors may contribute to immunomodulation and should be taken into consideration and studied when designing new bispecific reagents. For example, it may be possible to overcome a large synapse by improving affinity (and vice versa), or to overcome a small number of synapses by increasing avidity (and vice versa).

### New immune targets

Aptamers have emerged in recent years with affinity for two general classes of immune cell targets that are especially relevant for this review. The first set antagonize co-inhibitory receptors, for which a few aptamers have already been selected that target the more novel ICI targets such as TIM3 and LAG3.^53^^,^^55^ Many patients treated with conventional ICIs are either nonresponsive to therapy or acquire resistance. As noted by Nair and Elkord,^85^ co-blockade of immune checkpoints may be needed to overcome such resistance, suggesting potential value for therapies that can be used as adjuvants. Antagonizing novel co-inhibiting receptors, such as TIGIT, VISTA, B7-H3, BTLA, and CD73, is proving to be reliable at preventing CTLs from reaching exhaustion. This is exemplified by the various antibody therapies currently in clinical trials for advanced-stage tumors.^91^^,^^92^ To date, however, no published bsApts have targeted co-inhibition. New aptamers that target these co-inhibiting receptors or their ligands could provide an adjuvant therapy to improve immunomodulation of cancer. With a significant number of patients who receive ICI therapy experiencing grade 3–4 irAEs, it may be strategic to explore options that are less immunogenic and that would target Teff function to the TME.^159^ Both of these goals can be achieved using bsApts.

The second set of aptamers agonize co-stimulatory receptors (e.g., 4-1BB, CD28), and many of the available bsApts fall into this category. While these aptamers have been shown to improve T cell effector function, decrease tumor growth, and potentiate vaccination, they are inherently limited by the need for neoantigen presentation on MHC I. CAR T cells and some BiTEs circumvent the need for MHC I-mediated neoantigen presentation, which is part of what makes them such powerful and versatile immunomodulatory tools. Most BiTEs target and activate CD3ε, which, per se, can be enough to either induce or co-potentiate T cell activation. CAR T cells, on the other hand, achieve MHC I-independent T cell activation by introducing a genetically engineered receptor in which the extracellular portion exploits the TAA recognition activity of an antibody (often an scFv) and the intracellular portion mimics the signaling machinery of a TCR. Binding of a TAA to the receptor induces intracellular signaling pathways and T cell activation. In recent years, second- and third-generation CAR T cells have been engineered to improve and strengthen intracellular signaling.^160^ By extension, bsApts can be engineered to improve immunomodulation by targeting aptamers to CD3ε or by bringing CAR T cells in closer proximity to their TAAs.^43^^,^^72^^,^^73^ A potential limitation is that CD3 is also present on immunosuppressive cell types such as Tregs (albeit to a lesser extent), and a more ideal candidate for engaging T cells may yet be discovered.

### Altering the TME

If we continue to target co-stimulatory or inhibitory molecules as part of bsApts, it would be strategic to alter the TME to provide the ideal conditions for antigen-specific, immune-mediated cytotoxicity (e.g., increase the number of cells with co-stimulatory or inhibitory capabilities, such as CTLs, or improve tumor cell neoantigen presentation on MHC I). The TME describes the various cell types (e.g., lymphocytes, granulocytes, macrophages, fibroblasts, endothelial cells), signaling molecules (e.g., cytokines, chemokines, growth factors), extracellular matrix (ECM) proteins, and vascular components that make up the tumor environment.^161^^,^^162^ Getting tumors to express less immune-tolerant (neo)antigens could provide a powerful strategy for the recruitment of CTLs to the TME and for achieving immune-mediated prevention of tumor growth. The observations made by Pastor et al.^163^ show that improving tumor neoantigen presentation through the targeted knockdown of non-sense-mediated mRNA decay (NMD) indirectly alters the TME. However, other observations by Soldevilla et al.^42^ suggest that over-expression of neoantigens alone (induced by the same mechanism as that used by Pastor) may not always be sufficient to promote a potent anti-tumor immune response in some tumors. This was noted by the elevated levels of Foxp3 expression in lymphoma cells upon knockdown of SMG1 within the TME, suggesting high levels of the immunosuppressive Treg population. Nonetheless, these studies appear to point in the right direction. Obtaining a better understanding of the neoantigen characteristics that are presented in each of these models/studies may aid in discriminating which cell types are recruited to the TME. Other opportunities to alter the TME look at using combined therapies, either through the creation of multispecific aptamers or dual therapy, to prevent the accumulation of immunosuppressive cell types (such as MDSCs) or cytokines (such as IL-10 and TGFβ) or to induce the recruitment and activation of lymphocytes. An example of this dual approach is detailed above, in which inhibiting NMD was combined with the anti-4-1BB agonistic aptamers to improve immune-related tumor cell cytotoxicity.^163^

## Improving tumor cell recognition

### Neoantigen discovery: Differentiating healthy self from mutated self

Bispecific therapeutics depend upon an ability to differentiate between healthy self and mutated self to limit on-target/off-tumor side effects. While this can often be fulfilled by antigens of hematopoietic differentiation in various malignancies of the blood (e.g., CD19, BCMA, CD33), it can be extremely difficult to achieve with solid tumors, for which cancer cells typically contain few component parts (e.g., cell surface receptors) that are evolutionarily divergent from their homeostatic counterparts. Instead, these tumor cells often differ only in the *ratios* of component parts that are expressed normally. The impact of this paucity of unique markers is illustrated by the minimal progress made in solid tumor treatment compared with hematological malignancy treatment, despite innovative efforts to target them.^164^

The problem of differentiating healthy self from mutated self manifests on several fronts. For many bsApts and bsAbs, the tumor-targeting component usually has affinity to a cell surface molecule or receptor. Unfortunately, these targeted molecules are often also present on normal cells of similar origin, and are sometimes even expressed in high levels in other cell types,^109^^,^^165^ leading to unwanted on-target/off-tumor effects. Alternatively, some bsApts target stromal proteins,^77^^,^^78^ which has the advantage of being widely applicable to many tumor types.^166^^,^^167^ However, as with most tumor surface proteins, most stromal targets are also found outside of the TME. For example, VEGF and OPN are found in increased levels in breast cancer and GBM, but are also highly expressed in other tissues, such as those undergoing neuronal, cardiac, or bone repair.^78^^,^^122^^,^^168^^,^^169^

Another important factor in identifying ideal cell surface neoantigens is whether they remain on the surface or are rapidly internalized upon binding their natural ligands or the recognition module of the therapeutic reagent of choice. Internalization can be advantageous for intracellular delivery of therapeutic RNAs and peptides. However, target receptors must persist at the cell surface when formation of an immune synapse is needed to induce an anti-cancer effect. For example, PSMA is a membrane protein that is rapidly internalized into an endosomal compartment upon ligand binding.^115^^,^^120^ Pastor et al.^79^ had to mutate the domain responsible for internalization for the PSMA/4-1BB bsApt to exert its therapeutic effects. This suggests that cell surface receptors such as PSMA, EGFR, HER2, and various other RTKs and G protein-coupled receptors (GPCRs) that rapidly internalize, either constitutively or upon ligand/reagent binding, may be poor targets for immunomodulating bsApts unless they can be made to persist at the surface.^170^^,^^171^ These problems highlight the need to identify new TAAs. One approach is neoantigen discovery using cell-SELEX, in which whole cells serve as the selection target without prior knowledge of the surface features that will drive aptamer selection (reviewed in Pang et al.^172^ and Tawiah et al.^173^). A significant challenge of this method, however, is that clonal cancer cell lines are inherently heterogeneous, making it exceedingly difficult to find control cell populations that ensure effective subtraction of aptamer sequences that target *all* commonly expressed antigens.^9^^,^^174^^,^^175^

### Multispecific aptamers that recognize two or more tumor cell surface markers

Another opportunity to improve tumor specificity is to engineer the tumor-targeting aptamers so that multiple, simultaneous recognition events are required to form an effective synapse.^146^ A key tenet of this approach is to target antigenic combinations that are unique to a given tumor. This approach could not only improve specificity and limit toxic side effects but it could also provide additional mechanisms of tumor toxicity, in turn increasing the potency of the multispecific agent.^176^ For example, Zheng et al.^136^ created a non-immunomodulating bsApt that targeted CD44 and EpCAM, two tumor cell surface markers that are overexpressed in ovarian cancer and are associated with malignant ascites, chemoresistance, decreased survival, and epithelial-to-mesenchymal transition (EMT). The group noted that their bsApt increased cell lysis via apoptosis in various ovarian cancer cell lines *in vitro* and significantly suppressed tumor growth compared with monovalent aptamers *in vivo*. In principle, constructing a trispecific aptamer that added a T cell agonizing aptamer (e.g., CD28Apt7 or M12-23) into this apoptosis-inducing bsApt could make for an extremely potent and specific anti-cancer reagent.

The additional complexity of multispecific designs requires that the relevant variables, such as linker length, receptor localization, and valency, be further studied. For example, linker length played an important role when Wang and colleagues^176^ constructed a bsApt targeted to c-Met and TfR (or c-Met and nucleolin) to induce artificial protein-protein pairing in *cis* on the tumor cell surface. The artificial pairing of these receptors induced strong steric hinderance of c-Met, thereby preventing ligand (HGF) binding, receptor dimerization, downstream phosphorylation events, and subsequent cell migration. They noted, however, that the bsApt function was limited by linker length (ideal linker length 18–40 bp), with significantly diminished effects if the linker was too long (80 bp) or too short (9 and 12 bp). Receptor localization is another important consideration, as exemplified by the bsApt constructed by Miao and colleagues (targeting IGFIIR and c-Met) that induced receptor internalization.^144^ While increasing the internalization of certain tumor target receptors that drive distinct oncogenic signaling may prevent ligand binding, aberrant receptor signaling, and ultimately tumor growth, antigenic modulation and fast receptor internalization are not warranted when bsApts are used to form artificial synapses, as detailed in the previous section. Finally, while increasing the valency of tumor-targeting aptamers would likely increase tumor cell avidity, this effect could also improve recognition of non-malignant cells that still expressed the same combination of target receptors, albeit at lower levels. In addition, the impact of increasing bsApt valency and avidity on the immune-system-driven cytotoxicity is still largely unknown and understudied.^32^^,^^146^ General considerations during bispecific aptamer development are summarized in Figure 4.

## Improving efficacy and safety for a future in clinical trials

As of January 2022, 12 oligonucleotide therapies have been given regulatory approval from the FDA, including two mRNA-based vaccines against severe acute respiratory syndrome coronavirus-2 (SARS CoV-2) (one currently in emergency use authorization).^177^, ^178^, ^179^ Of the other 10, seven are single-stranded antisense oligonucleotides (ASOs), two are siRNAs, and one is a chemically modified RNA aptamer (pegaptanib/Macugen; targets and inhibits a VEGF isoform). While the number of proof-of-concept studies continues to increase, the number of successful clinical applications remains stagnant, suggesting that there is a translational bottleneck for oligonucleotide therapies. This can in part be attributed to the lack of attention given to improving the function and delivery of such proof-of-concept reagents. It can also be attributed to the fact that many reagents have been selected against murine targets (for ease of studying *in vivo*) and have minimal or no cross-reactivity with human homologs. This is especially true for proof-of-concept immunomodulating bsApts, such as those that target 4-1BB. However, the recent widespread acceptance of mRNA-based vaccines may portend accelerating interest in oligonucleotide therapies in general (and bsApts specifically). Thus, the field will need to place a major focus on addressing concerns related to successful translation from bench to bedside. To guide future studies on and improve the efficacy and safety of bsApts, we can use current FDA-approved oligonucleotide therapies, which have had to overcome various hurdles, including delivery (e.g., pharmacokinetics and pharmacodynamics), off-target interactions, and chemistry-dependent toxicity.

Very few studies on immunomodulating bsApts have explored the hurdles associated with FDA approval for oligonucleotide therapies. Most literature on immunomodulating bsApts has included both *in vitro* and *in vivo* studies, the majority of which focus on induction of immune cell activation, subsequent tumor cell lysis, and improved survival. Only two groups looked at non-specific T cell activation, and almost none looked at biodistribution beyond tumor tissue.^77^^,^^79^ Given that serious adverse events, poor circulation times, and high accumulation at the liver, kidneys, and spleen were some of the limiting factors of oligonucleotide therapies developed to date, it is imperative that these studies be added to experimental pipelines for bsApts.

ASOs are perhaps the most clinically developed oligonucleotide therapy, with dozens of new constructs entering clinical trials in the past few years.^179^^,^^180^ ASOs are 6–7 kDa amphipathic oligonucleotides that distribute broadly to many tissues *in vivo* and ultimately alter the expression of otherwise non-druggable targets such as transcription factors and other non-cell surface proteins. After binding their mRNA targets, ASOs can alter the mRNA's fate either via enzymatic degradation by RNase H1 recruitment or via occupancy-only methods to perturb splicing, translation, or miRNA modulation. Nearly every ASO in the clinical pipeline carries substantial covalent modifications, the most common of which are 2′-*O*-methyoxyethl (2′ MOE) and phosphorothioate (PS). The 2′ MOE modification improves pharmacokinetics and annealing affinity to the RNA target sequence, and it causes a broad reduction in class toxicities due to non-specific protein binding and immune stimulation. PS modifications improve pharmacokinetics and protein binding, and they support both enzymatic degradation of targeted RNA and occupancy-only mechanisms of action. 5′ PEGylation and 3′ inverted dT are often included to extend the circulating half-lives of ASOs. Similarly, most monovalent aptamers currently in the clinical pipeline carry additional modifications beyond 2′-FY, often including several specific sites containing 2′ OMe purines. In contrast, most studies of immunomodulating bsApts have used either unmodified nucleotides or only 2′-FY modifications and have not looked at medicinal chemistry approaches to improve nuclease susceptibility, serum-half-life, binding affinity, or biodistribution. Crooke et al.^180^ states that it is essential to define the chemistry of RNA-targeted oligonucleotides precisely, as subtle chemical modifications (e.g., 2′ MOE versus 2′ methoxy) can result in substantial changes in potency, pharmacokinetics, and generic chemical class effects. Therefore, it is critical that aptamers be subjected to the same scrutiny and that we test new bsApts using a similar (or new) set of chemical modifications.^29^^,^^34^

Finally, most candidate aptamer therapeutics that are effective in achieving their biochemical or biological endpoints nevertheless fail to advance through clinical development because they are deemed non-superior or inferior to existing therapies.^181^ This suggests a need to test novel bsApts in pre-clinical models in combination with and relative to conventional cancer therapies such as chemotherapy, radiation, and surgery in addition to unconventional cancer therapies such as vaccination. Using well-established murine and large-animal models that mimic clinical scenarios (e.g., autochthonous tumors and surgical resection) will also play an important role in this endeavor.^77^^,^^182^

## Conclusions and future directions

The ability to harness the power of immune cells and direct their action toward the TME is transforming cancer treatments. There is still much to learn about how we can use bsApts to manipulate the immune system and alter the TME to best guide these cells to perform their lytic actions. Many bsApts described in the literature were generated as proof-of-concept designs. It is time to recognize the therapeutic potential of these reagents and take the next steps to improve their clinical translation potential and ultimately move them into the clinical pipeline in a timely and safe manner. In this endeavor, it will be important to take lessons learned from other biologics that are already in this pipeline, such as bsAbs and other oligonucleotide-based therapeutics (ASOs and siRNAs). By better understanding the cell-cell junctions and artificial immune synapses created by these bsApts, we can design superior therapies that leverage the full power of the immune system. Combining such therapies with other standards of care in pre-clinical models will save patients the burden of unintended adverse events. Many novel opportunities also await, such as creating new multispecific reagents and combining them with various other immunomodulating therapies (e.g., aptamers, antibodies, ASOs) to increase efficacy, decrease cancer burden, and ultimately save lives.
